# Diversity and use of ethno-medicinal plants in the region of Swat, North Pakistan

**DOI:** 10.1186/1746-4269-9-25

**Published:** 2013-04-15

**Authors:** Naveed Akhtar, Abdur Rashid, Waheed Murad, Erwin Bergmeier

**Affiliations:** 1 Department of Botany, Islamia College University, Peshawar 25000, Pakistan; 2 Department of Vegetation Analysis and Phytodiversity, Albrecht von Haller Institute of Plant Sciences, Georg August University, Göttingen 37073, Germany; 3 Centre of Plant Diversity, University of Peshawar, Peshawar 25000, Pakistan; 4 Department of Botany, Kohat University of Science and Technology, Kohat 26000, Pakistan

**Keywords:** Ecosystem services, Ethnobotany, Medicinal plants, Miandam, Phytomedicine, Plant applications, Plant conservation, Vernacular plant names

## Abstract

**Background:**

Due to its diverse geographical and habitat conditions, northern Pakistan harbors a wealth of medicinal plants. The plants and their traditional use are part of the natural and cultural heritage of the region. This study was carried out to document which medicinal plant species and which plant parts are used in the region of Swat, which syndrome categories are particularly concerned, and which habitat spectrum is frequented by collectors. Finally, we assessed to which extent medicinal plants are vulnerable due to collection and habitat destruction.

**Methods:**

An ethnobotanical survey was undertaken in the Miandam area of Swat, North Pakistan. Data were collected through field assessment as well as from traditional healers and locals by means of personal interviews and semi-structured questionnaires.

**Results:**

A total of 106 ethno-medicinal plant species belonging to 54 plant families were recorded. The most common growth forms were perennial (43%) and short-lived herbs (23%), shrubs (16%), and trees (15%). Most frequently used plant parts were leaves (24%), fruits (18%) and subterranean parts (15%). A considerable proportion of the ethno-medicinal plant species and remedies concerns gastro-intestinal disorders. The remedies were mostly prepared in the form of decoction or powder and were mainly taken orally. Eighty out of 106 ethno-medicinal plants were indigenous. Almost 50% of the plants occurred in synanthropic vegetation while slightly more than 50% were found in semi-natural, though extensively grazed, woodland and grassland vegetation. Three species (*Aconitum violaceum*, *Colchicum luteum, Jasminum humile*) must be considered vulnerable due to excessive collection. Woodlands are the main source for non-synanthropic indigenous medicinal plants. The latter include many range-restricted taxa and plants of which rhizomes and other subterranean parts are dug out for further processing as medicine.

**Conclusion:**

Medicinal plants are still widely used for treatment in the area of Swat. Some species of woodlands seem to be adapted to wood-pasture, but vulnerable to overcollecting, and in particular to deforestation. It is suggested to implement local small-scaled agroforestry systems to cultivate vulnerable and commercially valuable ethno-medicinal woodland plants under local self-government responsibility.

## Introduction

Plants are an important source of traditional medicine for the treatment of various diseases [1]. It has been estimated that herbal medicines are used by more than 80% of the world’s population in developing countries to meet their primary healthcare needs [2]. In Pakistan, the available modern healthcare services are either insufficient or inaccessible and unaffordable to the majority of people. In addition, due to illiteracy and poverty most of the population is dependent on traditional phytomedicine to cure various ailments. As the country has diverse socio-economic, ethnic, linguistic and cultural areas, as well as unique biodiversity, copious knowledge of indigenous medicinal plants and their use in treating human ailments might reasonably be expected. More than 10% of the national flora of Pakistan (600–700 plant species) are used for medicinal purposes [3]. Phytomedicinal research in Pakistan is a recent activity and the documentation of ethnomedicinal plant knowledge and its applications are ongoing [3-6]. The loss of precious medicinal plant wealth due to overgrazing, agricultural expansion, environmental degradation, acculturation and deforestation, enhanced by population pressure and poverty, has been reported by various researchers [3,7-10] but information on which medicinal plant species in particular are vulnerable, and why, is lacking.

Traditional resources of medicinal plants from Chitral, North Pakistan, have been evaluated [7,11]. Several studies exist on the ethnomedicinal use of plants in different regions of Swat, North Pakistan [8,12-15]. In an ethno-medicinal study from the valley of Miandam, Swat, a total of 179 plant species have been listed [16], with medicinal use reported for 27 plants, but without reference to local names, habitats, and which parts of these plants are used. Recording the indigenous knowledge of medicinal plants is an urgent task. Traditional knowledge is usually passed verbally from generation to generation, and valuable information about medicinal plants is easily lost if not preserved in written form. The main objective of the present study was therefore to survey and to document the scattered indigenous knowledge of medicinal plants of the Miandam valley as basis for future phytochemical and pharmacological studies. Moreover, and for the first time in any region of Pakistan, the medicinal plants of the study area are classified according to biological and distributional properties as well as ecological preference. It is essential to know where and in which habitats ethno-medicinal plants occur, as such knowledge is a prerequisite to identify vulnerable plant species susceptible to collecting or habitat change.

### Study area

The valley of Miandam, Swat, is a well-known summer resort in northern Pakistan. Located about 50 km northeast of Saidu Sharif, the valley lies between 35° 4' N and 72° 29-32' E in the mountain range of Hindu Raj [17]. The study area ranges between 1400 to 3900 m a.s.l. It is a narrow valley with a number of gorges, bounded on the north, east and south by high mountains. Its western boundary is the river Swat. Gujars (Indian Aryans) and Yousafzai (Pakhtoon) are the two main tribes residing in the area. Their main source of income is agriculture (nearly 41%) [18] and most of the population of the study area is directly or indirectly engaged in it. Miandam is a mountainous region and the cultivated land is insufficient for subsistence. Additional sources of income are daily wages and salaries (20%), foreign and domestic remittances (17%), forest products including medicinal plants (12%) and other professions (10%). Findings from [19] reveal that 59% of the households in north-western Pakistan derive their income from the forests.

Due to its considerable variation in altitude, temperature, topography, soil type and moisture, the vegetation of Miandam Valley can be classified into a series of altitudinal belts, namely dominated by *Olea ferruginea* and *Quercus oblongata* (submontane)*, Pinus wallichiana, Abies pindrow, Picea smithiana* and *Quercus semecarpifolia* (montane), and alpine-subalpine flora, respectively [16]. See also the vegeation maps of the northern Pakistan regions of Chitral and Hunza [20,21].

## Methods

Regular field surveys were carried out in the Miandam valley from September 2010 through July 2011 in order to document the habitats and indigenous uses of ethno-medicinal plants of the valley. The surveys were carried out at different seasons so as to obtain identifiable plants and multiple information and also to cross-check the information provided by the local informants during earlier visits. We interviewed a small group of chiefly elder people of both Gujars and Yousufzai tribes who were highly esteemed in their societies due to their sound knowledge of medicinal plants. Structured questionnaires, formal and informal interviews and participatory observations were used to inquire about vernacular names, used plant parts and the process of remedy preparation. We did not encounter controversial issues among the informants but commonly received complementary information. Moreover, for each plant species growth forms (tree, shrub, woody climber, perennial herb, annual or biennial herb), plant status (indigenous, established alien, cultivated), abundance in the area (common, scattered, rare) and habitat preferences (arable fields, ruderal sites, wetland, woodland, mountain grassland) were recorded. Voucher specimens were identified using relevant standard literature [22-25] and submitted to the Herbarium PUP at the Department of Botany, University of Peshawar. Plant nomenclature was updated using the World Checklist of Selected Plant Families (http://apps.kew.org/wcsp/home.do) and The Plant List (http://www.theplantlist.org/). Family assignation in this paper follows the Flora of Pakistan [25].

## Results and discussion

### Plant diversity, use and applications

A total of 106 ethno-medicinal plant species belonging to 96 genera and 54 plant families were recorded. The plants have been used to treat a wide range of diseases from simple headache to complex disorders of kidney and liver. The results are presented in Table 1 with family names in alphabetical order, taxon name, local name, parts used, medicinal use, growth form, plant status, frequency and habitat preference. Perennial herbs were the most common growth form among medicinal plants (43%), followed by annuals and biennials (23%), shrubs (16%) and trees (15%) As far as documented the use of herbs for remedy preparation in the study area is in consistence with other studies [11,26-40].

**Table 1:** Medicinal plants of the Miandam area with their medicinal properties, and biological, ecological and chorological characteristics

**Plant family**	**Taxon name**	**Local name**	**Parts used**	**Medicinal uses, remedies**	**Growth form**	**Plant status**	**Frequency**	**Habitat**
Amaranthaceae	*Amaranthus viridis*	Chalvaray	Leaves	Leaf extract is emollient, also used for curing cough and asthma.	Annual	Indigenous	Common	Ruderal
Anacardiaceae	*Pistacia chinensis*	Shnai	Insect galls, leaves and bark	Powdered insect galls, bark and leaves are topical antiseptic, also for curing jaundice and liver diseases.	Tree	Established alien	Scattered	Woodland
Apiaceae	*Bupleurum longicaule*	Gillo	Whole plant	Powdered plant is mixed with milk and used as laxative	Perennial	Indigenous	Common	Woodland
Apiaceae	*Coriandrum sativum*	Dhanyal	Whole plant	Stimulant and carminative	Annual	Cultivated	Common	Arable
Apiaceae	*Foeniculum vulgare*	Kaga vanalay	Fruit	Powdered fruit is mixed with sugar, taken with a cup of milk for curing urinary problems (dysuria); dry fruits are carminative and laxative	Annual	Cultivated	Common	Arable
Apiaceae	*Pimpinella diversifolia*	Watani kaga	Fruit	Powdered fruits are carminative	Perennial	Indigenous	Scattered	Woodland
Apiaceae	*Heracleum candicans*	Kadu panra	Root	Decoction of root against colic and asthma	Perennial	Indigenous	Scattered	Wetland
Araceae	*Arisaema jacquemontii*	Marjarai	Rhizome	Rhizome bolus is given orally to livestock for respiratory problems	Perennial	Indigenous	Scattered	Woodland
Araliaceae	*Hedera nepalensis*	Prewata	Leaves	Juice from leaves for curing diabetes, also considered as blood purifier	Woody climber	Indigenous	Common	Woodland
Asclepiadaceae	*Periploca aphylla*	Barara	Stem, fruits	Milky juice of stem and fruit applied to swellings; stem latex as antimycotic for curing dermatitis in livestock	Shrub	Indigenous	Common	Ruderal
Asteraceae	*Artemisia scoparia*	Jaukay	Shoot and seeds	Respiratory stimulant, anthelmintic, purgative and against earache	Biennial	Indigenous	Common	Woodland
Asteraceae	*Cichorium intybus*	Han	Root	Decoction of fresh root for treatment of fever	Perennial	Indigenous	Common	Ruderal
Asteraceae	*Echinops echinatus*	Ghwand Saray Ghanowala	Root	Powdered root applied to wounds of cattle for killing maggots; also to kill lice	Perennial	Indigenous	Scattered	Wetland
Asteraceae	*Launaea procumbens*	Shauda pai	Leaves	Mixture of powdered leaves with sugar to enhance lactation in livestock	Perennial	Indigenous	Common	Ruderal
Asteraceae	*Sonchus asper*	Shauda pai	Shoot	Shoots fed to livestock for enhancing lactation	Annual	Indigenous	Common	Ruderal
Asteraceae	*Taraxacum* sp.	Ziar gulai	Leaves and roots	Grinded leaves are tonic, root decoction against kidney and liver disorders	Perennial	Indigenous	Common	Ruderal
Asteraceae	*Xanthium strumarium*	Ghishkay	Leaves	Leaf decoction recommended in malarial fever	Annual	Indigenous	Common	Ruderal
Berberidaceae	*Berberis lycium*	Kwaray	Root bark	Dried root bark given orally as body tonic	Shrub	Indigenous	Scattered	Woodland
Berberidaceae	*Podophyllum hexandrum*	Kakora	Rhizome	Powdered rhizome used to cure liver diseases	Perennial	Indigenous	Scattered	Woodland
Boraginaceae	*Cynoglossum lanceolatum*	Gat gul	Whole plant	Powdered plant taken with a decoction of *Coriandrum sativum* fruits as laxative	Perennial	Indigenous	Common	Woodland
Boraginaceae	*Onosma hispida*	Khwaga abai	Root	Used to color mustard oil which is applied for smoothing hair	Perennial	Indigenous	Common	Ruderal
Brassicaceae	*Brassica campestris*	Sharshum	Seeds	Oil, extracted from seeds, is used as ointment, for massage of body and hair	Annual	Cultivated	Common	Arable
Brassicaceae	*Brassica campestris* var. *rapa*	Tepar	Leaves, roots	Against stomachache and ulcer problems	Annual	Cultivated	Common	Arable
Brassicaceae	*Capsella bursa-pastoris*	Bambesa	Leaves and seeds	Paste of fresh leaves with milk for curing diarrhea; seeds are stimulant and diuretic	Annual	Indigenous	Common	Ruderal
Brassicaceae	*Nasturtium officinale*	Talmera	Young shoot	Young shoot against constipation and stomachache	Perennial	Indigenous	Common	Wetland
Buxaceae	*Sarcococca saligna*	Ladanr	Leaves	Heated in mustard oil and applied to muscular pain; infusion of leaves orally for rheumatism	Perennial	Indigenous	Common	Woodland
Cannabaceae	*Cannabis sativa*	Bang	Leaves	Leaves in bandage for wound healing; powdered leaves as anodyne, sedative, tonic and narcotic; juice added with milk and nuts as a cold drink (“Tandai”) generating a pleasant excitement; “Charas” is also prepared from it	Annual	Indigenous	Common	Arable
Caprifoliaceae	*Sambucus wightiana*	Benakai	Leaves, fruits and flowers	Poultice from leaves and flowers to treat burns and rheumatism; berries are purgative and used in dropsy	Shrub	Indigenous	Rare	Woodland
Caprifoliaceae	*Viburnum grandiflorum*	Ghuz meva	fruit	Fresh fruit is eaten to cure stomach problems	Shrub	Indigenous	Common	Woodland
Caryophyllaceae	*Arenaria griffithii*	Kinar	Shoots	Dried shoot powder with honey after meal as antispasmodic	Perennial	Indigenous	Common	Woodland
Caryophyllaceae	*Silene vulgaris*	Matorangay	Shoot	Shoot against stomachache and as emollient	Perennial	Indigenous	Common	Woodland
Caryophyllaceae	*Stellaria media*	Oulalai	Whole plant	Decoction is considered as purgative	Annual	Indigenous	Common	Arable
Chenopodiaceae	*Chenopodium album*	Sarmay	Whole plant	Dried powdered plant considered as carminative and diuretic agent	Annual	Indigenous	Common	Ruderal
Clusiaceae	*Hypericum perforatum*	Shin chai	Shoot	Used as diuretic and its tea is stimulant and analgesic	Perennial	Indigenous	Scattered	Woodland
Convolvulaceae	*Convolvulus arvensis*	Prewatai	Whole plant	Purgative, also applied in skin disorders	Perennial, climber	Indigenous	Common	Arable
Cuscutaceae	*Cuscuta reflexa*	Zelai	Whole plant	Decoction for urine control, diabetes and blood purification; plant extract used as anti-lice	Perennial, climber	Established alien	Scattered	Arable
Dioscoreaceae	*Dioscorea deltoidea*	Kanis zelai	Rhizome	Powdered rhizome mixed with powdered root of *Berberis lycium*, the mixture is used for treatment of jaundice and ulcers	Perennial, climber	Indigenous	Scattered	Woodland
Ebenaceae	*Diospyros kaki*	Sur amlok	Ripe fruits	Laxative	Tree	Cultivated	Common	Arable
Ebenaceae	*Diospyros lotus*	Tour amlok	Dried ripe fruits	Carminative, purgative and causing flatulence; boiled in milk and taken against constipation and dysentery	Tree	Cultivated	Common	Arable
Elaeagnaceae	*Elaeagnus umbellata*	Ghanum ranga	Flowers, leaves	Decoction of flowers used twice a day to cure heart diseases; decoction of leaves against cough; mature raw seeds eaten as vitamin C source	Shrub	Indigenous	Rare	Woodland
Euphorbiaceae	*Euphorbia wallichii*	Shangla	Whole plant	Dried leaves and seeds given to children in bowel complains; plant juice against ringworm	Perennial	Indigenous	Common	Woodland
Euphorbiaceae	*Ricinus communis*	Harhanda	Seeds	Seed oil demulcent and to evacuate bowels in children	Shrub	Established alien	Scattered	Ruderal
Fabaceae	*Indigofera heterantha*	Ghwarija	Root and leaves	Dried powdered root taken with glass of water against scabies; leaves against stomach problems	Shrub	Indigenous	Common	Woodland
Fabaceae	*Lathyrus aphaca*	Korkamanai	Seed	Decoction of the seed 3 times a day for wound healing	Annual	Indigenous	Scattered	Arable
Fabaceae	*Lotus corniculatus*	Fateh khana	Whole plant	Decoction of dried powdered plant with ghee or boiled water against sexual debility and backache	Perennial	Indigenous	Scattered	Woodland
Fagaceae	*Quercus oblongata*	Banj	Fruit	Powdered fruits in urinary infection	Tree	Indigenous	Common	Woodland
Fagaceae	*Quercus floribunda*	Tour banj	Fruit	Powdered fruits for treating gonorrhea and urinary disease	Tree	Indigenous	Common	Woodland
Fumariaceae	*Corydalis stewartii*	Mamera	Floral shoot	Decoction of floral shoot to cure eye diseases	Biennial	Indigenous	Scattered	Mountain grassland
Geraniaceae	*Geranium wallichianum*	Srazela	Root	Root decoction with pods of *Pistacia chinensis* to treat cough and fever and urinary complaints	Perennial	Indigenous	Common	woodland
Hippocastanaceae	*Aesculus indica*	Jawaz	Seeds and bark	Fruits are anthelmintic and given to horses in colic; plant oil externally used against rheumatism; nuts against colic and to cure chest diseases in horses, donkeys and mules	Tree	Indigenous	Scattered	Woodland
Juglandaceae	*Juglans regia*	Ghwaz	Fruit, bark, leaves	Dried fruit mixed with coconut and honey used as tonic; bark (locally called Dandasa) for cleaning and sparkling of teeth; decoction of leaves against eczema and intestinal worms	Tree	Cultivated	Common	Arable
Lamiaceae	*Ajuga bracteosa*	Booti	Whole plant	Locally, decoction of the plant or its powder swallowed with water before breakfast for the treatment of throat sore, internal colic, purifying blood and epilepsy; decoction for curing jaundice and hypertension	Perennial	Indigenous	Common	Ruderal
Lamiaceae	*Mentha spicata*	Podina	Leaves and stem	Carminative	Perennial	Cultivated	Common	Arable
Lamiaceae	*Mentha royleana*	Valenay	whole plant	Decoction of leaves for treatment of diarrhea in children; powdered plant mixed with sugar for prevention of vomiting and dyspepsia	Perennial	Indigenous	Common	Ruderal
Lamiaceae	*Nepeta cataria*	Pisho botai	Flowers and leaves	Dried leaves and flowering tops carminative	Perennial	Indigenous	Scattered	Mountain grassland
Lamiaceae	*Otostegia limbata*	Spin azghai	Whole plant	Juice of leaves applied to gums for treatment of gum problem in children; dried powder of plant is used in jaundice	Perennial	Indigenous	Common	Woodland
Lamiaceae	*Isodon rugosus*	Spearkai	Leaves	Dried leaves put in mouth as remedy for toothache	Shrub	Indigenous	Common	Woodland
Lamiaceae	*Origanum vulgare*	Shamakay	Whole plant	Diuretic and against toothache and earache	Perennial	Indigenous	Common	Woodland
Lamiaceae	*Salvia lanata*	Spera botai	Leaves	Paste of leaves applied to toes laceration in hot and moist season	Perennial	Indigenous	Scattered	Woodland
Lamiaceae	*Salvia moorcroftiana*	Kherghwag	Leaves	*Brassica campestris* oil applied to fresh leaves tied round for healing of wounds	Perennial	Indigenous	Common	Ruderal
Lamiaceae	*Thymus linearis*	Chi botai	Shoots	Tea of shoots advised for treating pain and fever	Perennial	Indigenous	Common	Mountain grassland
Liliaceae	*Allium sativum*	Ouga	Bulb and leaves	Boiled and the cooled extract administered against diarrhea, dysentery and for lowering blood pressure; bulbs stimulant; leaves diuretic, aphrodisiac and expectorant; antiseptic; juice applied to soothe irritation caused by scorpion and hornet stings	Perennial	Cultivated	Common	Arable
Liliaceae	*Allium cepa*	Piaz	Bulb and leaves	Bulbs stimulant; leaves diuretic, aphrodisiac and expectorant; also antiseptic and juice applied to soothe irritation caused by scorpion and hornet sting; Mountaineers have it with them while crossing high altitude passes as it enhances the intake of oxygen	Perennial	Cultivated	Common	Arable
Liliaceae	*Colchicum luteum*	Qaimat guallay	Whole plant	Blood purifier, laxative and aphrodisiac; fried corms are used for joints pain	Perennial	Indigenous	Rare	Mountain grassland
Liliaceae	*Polygonatum multiflorum*	Noorealam	Rhizome	Rhizome infusion against dysentery; referred aphrodisiac	Perennial	Indigenous	Scattered	Woodland
Liliaceae	*Polygonatum verticillatum*	Noorealam	Rhizome	Against rheumatism and as aphrodisiac	Perennial	Indigenous	Scattered	Woodland
Malvaceae	*Abelmoschus esculentus*	Bhindi	Fruits	Emollient, demulcent and diuretic	Annual	Cultivated	Scattered	Arable
Meliaceae	*Melia azedarach*	Tora bakyana, shandai	Fruits, shoots, bark, leaves	Dried, crushed fruits against gastric trouble, fever and cough; dry leaves mixed with wheat flour used as anthelmintic in livestock; decoction of the bark considered anti-allergic; extraction of leaves used by women against head lice; leaves, young branches or fermented fruits are given as carminative to cattle, when belly is swollen through gas accumulation due to overeating	Tree	Established alien	Scattered	Woodland
Moraceae	*Ficus palmata*	Inzer	Flowers and fruits	Fresh floral parts as demulcent; juice extracted from fruit as expectorant	Tree	Cultivated	Common	Arable
Moraceae	*Morus alba*	Toot	Fruit	Fruit to treat constipation and cough	Tree	Indigenous	Common	Arable
Oleaceae	*Jasminum humile*	Rambil chambil	Roots and flowers	Powdered roots as anthelmintic and diuretic; juice extracted from flowers against skin diseases, headache and mouth rash	Shrub	Indigenous	Rare	Woodland
Oleaceae	*Olea europaea*	Khona	Leaves	Decoction of leaves as gargle considered as remedy for toothache, mouth and gum diseases	Tree	Cultivated	Scattered	Arable
Oxalidaceae	*Oxalis corniculata*	Tarukey	Whole plant	Decoction of plant to enhance digestion	Annual	Indigenous	Common	Ruderal
Paeoniaceae	*Paeonia emodi*	Mamekh	Rhizome	Powdered rhizome with milk to cure backache and general weakness	Perennial	Indigenous	Scattered	Woodland
Papaveraceae	*Papaver somniferum*	Qashqash	Capsule, seeds	Capsules and seeds as narcotic; dried capsule to make tea for cough and fever	Annual	Indigenous	Scattered	Arable
Plantaginaceae	*Plantago lanceolata*	Jabai	Leaves	Leaves applied to treat bedsores, inflamed surfaces and candidiasis	Perennial	Indigenous	Scattered	Ruderal
Plantaginaceae	*Plantago major*	Ghwa jabai	Seeds, leaves	Leaves applied to treat bedsores and candidiasis	Perennial	Indigenous	Scattered	Ruderal
Platanaceae	*Platanus orientalis*	Chinar	Bark	Powdered bark taken orally to control diarrhea	Tree	Indigenous	Scattered	Woodland
Poaceae	*Avena sativa*	Jamdaray	Fruit	Fried in ghee and milk, the paste is considered as general body tonic and aphrodisiac	Annual	Cultivated	Common	Arable
Poaceae	*Cynodon dactylon*	Kabal	Whole plant	Decoction as blood purifier and to control nose bleed; chewed and placed on wound to stop bleeding and as topical anti-septic	Perennial	Indigenous	Common	Ruderal
Polygonaceae	*Rumex dentatus*	Shalkhay	Rhizome, leaves	Rhizome and leaves as poultice for wound healing	Annual	Indigenous	Common	Ruderal
Portulacaceae	*Portulaca oleracea* s.l.	Warkharae	Shoot	Shoot decoction against liver and kidney diseases	Annual	Cultivated	Common	Arable
Primulaceae	*Primula denticulata*	Mamera	Stem base	Infusion of young stem base ophthalmic	Perennial	Indigenous	Common	Woodland
Punicaceae	*Punica granatum*	Nangoray, Anar	Fruit	Dried fruit in bolus form for removal of intestinal helminths	Shrub	Cultivated	Scattered	Arable
Ranunculaceae	*Aconitum violaceum*	Zaharmora, Da Ghra Zahar	Rhizome	Rhizomes, wrapped in sheep or goat intestine and thoroughly boiled in milk; milk discarded and rhizomes crushed into powder, taken against rheumatism and arthritis; administering as such may cause death or mental problems if overdozed	Perennial	Indigenous	Rare	Woodland
Ranunculaceae	*Caltha alba*	Makan path	Leaves	Leaves laxative in nature	Perennial	Indigenous	Scattered	Wetland
Ranunculaceae	*Delphinium denudatum*	Jadwar	Rhizome	Rhizome powder with water to cure cough and fever	Perennial	Indigenous	Scattered	Woodland
Rosaceae	*Fragaria bucharica*	Da zmaki toot	Root, fruit	Powdered root useful in disease of urinary tract; fruits carminative and laxative	Perennial	Indigenous	Common	Woodland
Rosaceae	*Prunus armeniaca*	Khubanai	stem	Gum obtained from stem famed as anticancer	Tree	Cultivated	Common	Arable
Rosaceae	*Prunus domestica*	Alucha	Fruits	Fruit laxative	Tree	Cultivated	Common	Arable
Rosaceae	*Rosa moschata*	Gulab	Flowers	Decoction of flowers for curing stomach disorders	Shrub	Indigenous	Scattered	Woodland
Rosaceae	*Spiraea* spec.	Krachae	Flowers	Tea from its flowers to ease natal pain	Shrub	Indigenous	Common	Woodland
Rutaceae	*Skimmia laureola*	Nazar pana	Leaves	Burnt incense to expel evils and evil eyes; tea for indigestion, smoke considered as antiseptic	Shrub	Indigenous	Common	Woodland
Rutaceae	*Zanthoxylum armatum*	Dambara	Fruit	Fruits as antipyretic and for treating stomachache	Shrub	Indigenous	Scattered	Woodland
Saxifragaceae	*Bergenia stracheyi*	The Spinsar Gat Pana	Rhizome	Powdered rhizome with milk in the mornings as tonic	Perennial	Indigenous	Common	Woodland
Simaroubaceae	*Ailanthus altissima*	Backyanra	bark	Bark juice mixed with milk to cure dysentery and diarrhea	Tree	Established alien	Common	Arable
Solanaceae	*Atropa acuminata*	Bargak	leaves	Poultice of leaves against pain and rheumatism	Perennial	Indigenous	Scattered	Woodland
Solanaceae	*Capsicum annuum*	Marchakay	Fruits	Carminative	Annual	Cultivated	Common	Arable
Solanaceae	*Datura stramonium*	Batora	Leaves, seeds and flowers	poultice of flowers applied to wounds to reduce pain; seeds narcotic in nature	Annual	Indigenous	Common	Ruderal
Solanaceae	*Solanum nigrum*	Kachmacho	Leaves and fruit	Leave paste applied to treat skin inflammation, fruits against fever	Annual	Indigenous	Common	Ruderal
Solanaceae	*Solanum virginianum*	Marraghonay	Fruit	Decoction of fruit diuretic and anthelmintic	Perennial	Indigenous	Scattered	Ruderal
Solanaceae	*Withania somnifera*	Kotilal	Whole plant	Aphrodisiac	Shrub	Indigenous	Scattered	Ruderal
Thymelaeaceae	*Daphne mucronata*	Laighonai	Fruits, leaves	Poultice from fruits and leaves against rheumatism	Shrub	Indigenous	Common	Woodland
Ulmaceae	*Celtis australis*	Tagha	Fruits, bark	Fruits against colic and amenorrhea; bark decoction as anti-allergic	Tree	Indigenous	Scattered	Woodland
Urticaceae	*Debregeasia saeneb*	Ajlai	Leaves	Fresh ground leaves in paste form for blistered feet	Shrub	Indigenous	Common	Woodland
Verbinaceae	*Verbena officinalis*	Shamakai	Whole plant	Decoction is anti-malarial	Perennial	Indigenous	Common	Ruderal

Ninety-nine of the species (93%) are used for human ailments, three species (3%) for livestock cure and four (4%) to treat both human and livestock ailments. No less than 44 plant species were used to treat gastro-intestinal disorders such as dyspepsia, dysentery and stomach-ache followed by the treatment of dermatological diseases with more than 25 herbal remedies. Ten species were used against skeleto-muscular complaints like rheumatism, backache and muscular pain. Sixteen species were used to cure respiratory problems such as cough and asthma, fourteen for urinary complaints, twelve for cardio-vascular complaints and circulatory diseases, twelve to treat fever and headache, eleven for genital and sexual diseases, six for dental problems, six for ear, nose, throat (ENT) and eyes diseases, two for nerve disorders, one species (*Spiraea* spec.) was used to ease childbirth, and eighteen species for other purposes (wounds, cuts, narcotic, tonic, anticancer and tumor) (Table 2). The leaves of *Skimmia laureola* are used for spiritual purposes.

**Table 2:** List of ethno-medicinal plants applied with different syndromes

**Syndrome category**	**Plants**
**Gastrointestinal disorders**	*Aesculus indica, Ailanthus altissima, Ajuga bracteosa, Allium sativum, Artemisia scoparia, Brassica campestris var. rapa, Bupleurum longicaule, Capsella bursa-pastoris, Caltha alba, Celtis australis, Capsicum annuum, Chenopodium album, Colchicum luteum, Convolvulus arvensis, Coriandrum sativum, Cynoglossum lanceolatum, Dioscorea deltoidea, Diospyros kaki, Diospyros lotus, Euphorbia wallichii, Foeniculum vulgare, Fragaria bucharica, Heracleum candicans, Hypericum perforatum, Indigofera heterantha, Jasminum humile, Melia azedarach, Mentha spicata, Mentha royleana, Nasturtium officinale, Nepeta cataria, Oxalis corniculata, Pimpinella diversifolia, Plantago major, Platanus orientalis, Polygonatum verticillatum, Prunus domestica, Punica granatum, Ricinus communis, Rosa moschata, Sambucus wightiana, Skimmia laureola, Solanum virginianum, Stellaria media, Viburnum grandiflorum, Zanthoxylum armatum*
**Dermatological and topical diseases**	*Abelmoschus esculentus, Allium cepa, Allium sativum, Amaranthus viridis, Brassica campestris, Celtis australis, Convolvulus arvensis, Cuscuta reflexa, Cynodon dactylon, Datura stramonium, Debregeasia saeneb, Echinops echinatus, Euphorbia wallichii, Indigofera heterantha, Jasminum officinale, Juglans regia, Melia azedarach, Onosma hispida, Periploca aphylla, Pistacia chinensis, Plantago lanceolata, Plantago major, Salvia lanata, Sambucus wightiana, Silene vulgaris, Skimmia laureola, Solanum nigrum*
**Respiratory illness**	*Abelmoschus esculentus, Allium cepa, Allium sativum, Amaranthus viridis, Arisaema jacquemontii, Arenaria griffithii, Artemisia scoporia, Delphinium denudatum, Elaeagnus umbellata, Ficus palmata, Geranium wallichianum, Heracleum candicans, Melia azedarach, Morus alba, Papaver somniferum, Ricinus communis*
**Skeleto-muscular problems**	*Aesculus indica, Aconitum violaceum, Atropa acuminata, Colchicum luteum, Daphne mucronata, Lotus corniculatus, Paeonia emodi, Polygonatum verticillatum, Sambucus wightiana, Sarcococca saligna*
**Cardio-vascular complaints and circulatory diseases**	*Ajuga bracteosa, Allium sativum, Colchicum luteum, Cuscuta reflexa, Dioscorea deltoidea, Elaeagnus umbellata, Hedera nepalensis, Otostegia limbata, Pistacia chinensis, Podophyllum hexandrum, Portulaca oleracea, Taraxacum* spec.
**Fever, headache, analgesic**	*Cichorium intybus, Delphinium denundatum, Geranium wallichianum, Hypericum perforatum, Jasminum humile, Melia azedarach, Papaver somniferum, Solanum nigrum, Thymus linearis, Verbena officinalis, Xanthium strumarium, Zanthoxylum armatum*
**Urinary complaints**	*Abelmoschus esculentus, Allium cepa, Allium sativum, Capsella bursa-pastoris, Chenopodium album, Cuscuta reflexa, Foeniculum vulgare, Fragaria vesca, Hypericum perforatum, Portulaca oleracea, Quercus oblongata, Quercus floribunda, Solanum virginianum, Taraxacum* spec.
**Dental problems**	*Isodon rugosus, Juglans regia, Olea europaea, Origanum vulgare, Otostegia limbata, Rumex dentatus*
**ENT complaints**	*Ajuga bracteosa, Artemisia scoporia, Corydalis stewartii, Origanum vulgare, Primula denticulata*
**Nerve disorders (anodyne, epilepsy, sedative)**	*Ajuga bracteosa, Cannabis sativa*
**Genital and sexual diseases**	*Allium cepa, Allium sativum, Avena sativa, Celtis australis, Colchicum luteum, Geranium wallichianum, Lotus corniculatus, Polygonatum multiflorum, Polygonatum verticillatum, Quercus dilatata, Withania somnifera*
**Others (wounds, cuts, narcotic, tonic, tumor, anticancer and stimulant)**	*Allium cepa, Allium sativum, Avena sativa, Berberis lycium, Bergenia stracheyi, Cannabis sativa, Capsella bursa-pastoris, Coriandrum sativum, Cynodon dactylon, Datura stramonium, Juglans regia, Lathyrus aphaca, Paeonia emodi, Papaver somniferum, Periploca aphylla, Prunus armeniaca, Salvia moorcroftiana, Taraxacum* spec.
**Delivery**	*Spiraea* spec.

A single plant species may be used to cure several human ailments (Table 2). Some of the remedies were prepared by combining different plants such as the powdered rhizome of *Dioscorea deltoidea* mixed with powdered root of *Berberis lycium* for the treatment of jaundice and ulcers. Similarly, root decoction of *Geranium wallichianum* with pods of *Pistacia chinensis* was used for curing urinary complaints, cough and fever. According to traditional healers, complex medicines of two or more plant species are more potent than those prepared with single species. This has been attributed to interactive effects of the plants [41]. The most common medicinal recipe preparation was in powder form followed by decoction, infusion, juices, poultice and paste.

The traditional healers and local herbalists of the region usually utilize every part of the plant. However, the use of a particular plant part depends on the plant habit and user’s needs. The most frequently used plant parts in the preparation of herbal remedies were leaves (29%), followed by fruit (18%), roots and rhizomes (17%), and whole plants (7%). Seeds (9%), flowers (8%), bark (7%), bulbs (2%), capsules, floral shoots and insect galls (1% each) have also been used. The use of specific plant parts suggests that these parts have strongest medicinal properties but it needs biochemical analysis and pharmaceutical screening to cross-check the local information. Our findings of the frequent use of green leaves in the preparation of remedies corroborate the results of [42-46].

Different liquids such as water, juices, sugar, tea, honey, mustard oil, *desi ghee* (butter) and milk are mixed with plants or plant parts during the preparation of the remedies. The prepared remedies are mostly administered orally (77%), less frequently dermally (10%) or both orally and dermally (12%). Only 1% is administered through ears or eyes.

### Habitats and conservation of ethno-medicinal plants

Eighty-two out of 106 medicinal plants are indigenous to the area while the others are cultivated (19) or established alien plants (5). The latter groups are of no conservation concern as they are common (17) or scattered (7) in the study area. Also among the indigenous medicinal plants the majority of species is common (59%) or scattered (35%) in the area, thus neither of immediate conservation concern. Only five medicinal plant species (6%) are rare in the study area: *Aconitum violaceum*, *Colchicum luteum*, *Elaeagnus umbellata*, *Jasminum humile* and *Sambucus wightiana*. *Sambucus* and *Elaeagnus* are woodland shrubs of which leaves and fruits or leaves and flowers, respectively, are collected for medicinal purposes. Since this kind of harvesting is non-destructive, the rarity of the shrub species is apparently not caused by overcollection. In contrast, populations of *Aconitum violaceum*, *Colchicum luteum and Jasminum humile* may be harmed since rhizomes, corms or whole plants are collected, respectively. In these cases, plant populations should be monitored to avoid overcollection.

The synanthropic flora (i.e., occurring in arable fields or ruderal sites) contains a high proportion of the ethno-medicinal plants. Slightly under 50% (51) out of the 106 ethno-medicinal plant species occur in man-made habitats (in arable fields 27 species, most of which being cultivated; another 24 in ruderal sites). Since they can be expected to grow abundantly in or near settlements, or are even cultivated and harvested, they may be collected without much effort, and in suitable quantities. Slightly more than 50% (55) of the ethno-medicinal plant species encountered in the study area occur in semi-natural habitats (though extensively grazed or otherwise used). Most species of the latter group (47) occurred in different kinds of woodland, while only few occur in wetlands (4) and mountain grasslands (4). Mountain grassland medicinal plants known in the Miandam valley comprise *Colchicum luteum*, *Corydalis stewartii*, *Nepeta cataria* and *Thymus linearis*. Since Himalayan mountain floras are rich [45-47] and the local almost certainly contains more species of pharmaceutical value, we assume that the habitat is too remote and too difficult to access to be of much interest as a “medicinal plant hunting area” for the people in the Miandam valley.

Woodlands are the main source for non-synanthropic indigenous medicinal plants. They comprise 21 woody plants (apart from the climber *Hedera nepalensis*, seven trees and thirteen shrubs), two short-lived and 24 perennial herbs. Almost half of the perennial herbs are dug to collect the stem base (*Primula denticulata*) or chiefly the rhizomes (*Aconitum violaceum, Arisaema jacquemontii, Bergenia stracheyi, Delphinium denudatum, Dioscorea deltoidea, Paeonia emodi, Podophyllum hexandrum, Polygonatum multiflorum, Polygonatum verticillatum*). Except the latter two, these species are range-restricted taxa of Himalayan or narrower distribution. Due to their biochemical components they are largely unpalatable for livestock, hence fairly resistant under the widespread practice of wood-pasture, but may be vulnerable to overcollecting for medicinal purposes, although so far only *Aconitum violaceum* is considered rare in the study area. A currently more serious threat to the ethno-medicinal plant wealth of the woodlands as well as to the social and economic basis of the rural population in northern Pakistan is excessive timber exploitation leading to deforestation and habitat destruction.

## Conclusion

The Miandam valley in northern Pakistan is very rich in commercially and pharmaceutically important ethno-medicinal plant species. The locals, in particular traditional healers, have centuries-old knowledge regarding the uses of the plants, and the locals use these species in a traditional way for curing a wide spectrum of diseases. Few species were found to be vulnerable probably due to overcollection. Especially perennial woodland herbs with rhizomes are of conservation concern. The local inhabitants depend on plants for the treatment of diseases but not all are familiar with the proper collection, parts to be used, preservation and storage. In contrast, local traditional healers are familiar with proper collection and use of medicinal plants, and they should be involved in efforts of conservation and sustainable use of ethno-medicinal plant resources. In view of the outstanding importance and ecosystem services of woodlands and forests in northern Pakistan the currently widespread and uncontrolled deforestation is a serious threat both to ecological and social sustainability as well as to the long-term economic basis of the local population [19]. It is also a threat to the ethno-medicinal plant wealth. For purposes of plant conservation and to increase the locals’ income we suggest to cultivate vulnerable woodland medicinal plants of commercial value in newly designed and locally administered self-government agroforestry systems. Due to the specific habitat demands of many woodland plant species better results may be obtained through well managed agroforestry systems than in ex-situ sites [48].

## Competing interests

The authors declare that they have no competing interests.

## Authors’ contributions

NA carried out the field work, analyzed the data and drafted the manuscript. EB revised the whole manuscript and contributed to the editing and interpreting of the data. AR conceptualized and designed the study while WM helped in the initial drafting of the manuscript. All authors read and approved the final manuscript.
